# Application of metal-organic frameworks-based functional composite scaffolds in tissue engineering

**DOI:** 10.1093/rb/rbae009

**Published:** 2024-02-01

**Authors:** Xinlei Yao, Xinran Chen, Yu Sun, Pengxiang Yang, Xiaosong Gu, Xiu Dai

**Affiliations:** Key Laboratory of Neuroregeneration of Jiangsu and Ministry of Education, Co-Innovation Center of Neuroregeneration, Nantong University, Nantong 226001, China; Key Laboratory of Neuroregeneration of Jiangsu and Ministry of Education, Co-Innovation Center of Neuroregeneration, Nantong University, Nantong 226001, China; Key Laboratory of Neuroregeneration of Jiangsu and Ministry of Education, Co-Innovation Center of Neuroregeneration, Nantong University, Nantong 226001, China; Key Laboratory of Neuroregeneration of Jiangsu and Ministry of Education, Co-Innovation Center of Neuroregeneration, Nantong University, Nantong 226001, China; Key Laboratory of Neuroregeneration of Jiangsu and Ministry of Education, Co-Innovation Center of Neuroregeneration, Nantong University, Nantong 226001, China; Key Laboratory of Neuroregeneration of Jiangsu and Ministry of Education, Co-Innovation Center of Neuroregeneration, Nantong University, Nantong 226001, China

**Keywords:** metal-organic frameworks, function, composite scaffold, tissue engineering

## Abstract

With the rapid development of materials science and tissue engineering, a variety of biomaterials have been used to construct tissue engineering scaffolds. Due to the performance limitations of single materials, functional composite biomaterials have attracted great attention as tools to improve the effectiveness of biological scaffolds for tissue repair. In recent years, metal-organic frameworks (MOFs) have shown great promise for application in tissue engineering because of their high specific surface area, high porosity, high biocompatibility, appropriate environmental sensitivities and other advantages. This review introduces methods for the construction of MOFs-based functional composite scaffolds and describes the specific functions and mechanisms of MOFs in repairing damaged tissue. The latest MOFs-based functional composites and their applications in different tissues are discussed. Finally, the challenges and future prospects of using MOFs-based composites in tissue engineering are summarized. The aim of this review is to show the great potential of MOFs-based functional composite materials in the field of tissue engineering and to stimulate further innovation in this promising area.

## Graphical Abstract

**Figure:**
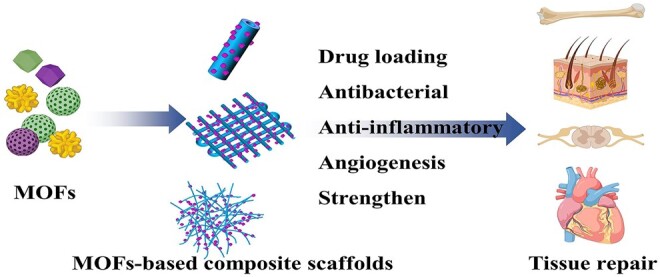

## Introduction

Tissue engineering scaffold materials are the material basis for reconstructing tissues and organs using tissue engineering technology and are thus one of the key factors in tissue engineering research [1–3]. With the rapid development of materials science and the increasing maturity of tissue engineering technology and theory, various scaffolds for tissue engineering have emerged [4–8]. Due to the complexity of the structure and composition of tissue, the specificity of various tissue functions and the limitations of biomaterial functions, it is difficult for a single biomaterial to meet the needs of tissue reconstruction. Therefore, functional composite scaffolds with special properties have attracted much attention [9–14]. Functional composite scaffolds usually consist of matrix materials and functional materials. Matrix materials determine the basic performance parameters of biomaterials, while functional materials determine the specialized performance parameters of biomaterials.

Among the wide variety of functional materials, metal-organic frameworks (MOFs), also known as porous coordination polymers, are a type of organic–inorganic hybrid material that has emerged in the field of tissue engineering in recent years. Compared with conventional organic and inorganic materials, MOFs have the advantages of compositional and structural diversity, porousness, ultrahigh specific surface area and excellent thermal and chemical stability [15–19]. Since the inception of MOFs, scientists have focused on their applications in the fields of gas storage [20–22], catalysis [23, 24], sensing [25–27] and drug delivery [28–31] due to the unique structure and properties of MOFs. With in-depth research on the properties of MOFs and the further expansion of their possible applications, an increasing number of studies on the application of MOFs in tissue engineering have been conducted in recent years, especially the last 5 years. It has been reported that some MOFs have desirable biocompatibility and biodegradability [32–35]. Some MOFs nanoparticles can enhance stem cell attachment, proliferation and differentiation [36, 37]. Furthermore, the functional groups of MOFs themselves, corresponding intelligent properties (such as pH responsiveness, photo responsiveness and thermal responsiveness), and degradation products can confer unique functions on modified materials, such as anti-inflammatory properties [38–40], antibacterial properties [41, 42] and angiogenesis promotion [43, 44]. In addition, the physical and chemical properties of biomaterials, such as roughness, porous structure, hydrophilicity and mechanical properties, will be altered by modification with MOFs, with the potential to promote cell adhesion, spreading, growth and proliferation [45–47]. Moreover, the high surface area and excellent porosity of nano-MOFs enable them to be used as carriers that can achieve the long-term stable release of small-molecule drugs or growth factors to improve therapeutic efficiency. In the past, such drug-loading functions were mostly applied to the targeted delivery of cancer drugs [28, 31, 48, 49]. In recent years, composite biomaterials, which are prepared by combining MOFs loaded with specific drugs with other biomaterials, have been used to fabricate tissue engineering scaffolds that can release anti-inflammatory, antibacterial or antioxidant drugs and growth factors to promote cell proliferation, growth or differentiation or tissue regeneration, facilitating efficient tissue repair [50–52].

However, there are few summaries of related work and discussions of the outlook of the field. Although there have been reviews related to the application of MOFs in tissue engineering and regenerative medicine, they mainly focus on the synthesis methods of MOFs, the key properties of biomaterials related to MOFs or their applications in specific tissues (bone and/or skin) [53–57]. The preparation of functional composite scaffolds based on MOFs, the specific functions of MOFs and the intrinsic mechanisms that achieve the corresponding functions have not been classified and summarized. In addition, many recent reports have not been addressed. In this review, the preparation methods of MOFs-based composite scaffolds for tissue engineering are classified, and the specific functions of MOFs themselves in composites and the intrinsic mechanisms of these functions are summarized. Then, the applications of MOFs-based composites in different tissues are presented. Finally, the current research status of MOFs-based composite scaffolds is summarized, and future development directions are anticipated. The purpose of this article is to summarize the existing research on functional composite scaffolds based on MOFs to provide researchers with a more comprehensive understanding of the research progress. It is also hoped that researchers will be inspired with more valuable ideas in this field.

## Construction methods of composite scaffolds based on MOFs for tissue engineering

### *In situ* growth

**Figure 1.**
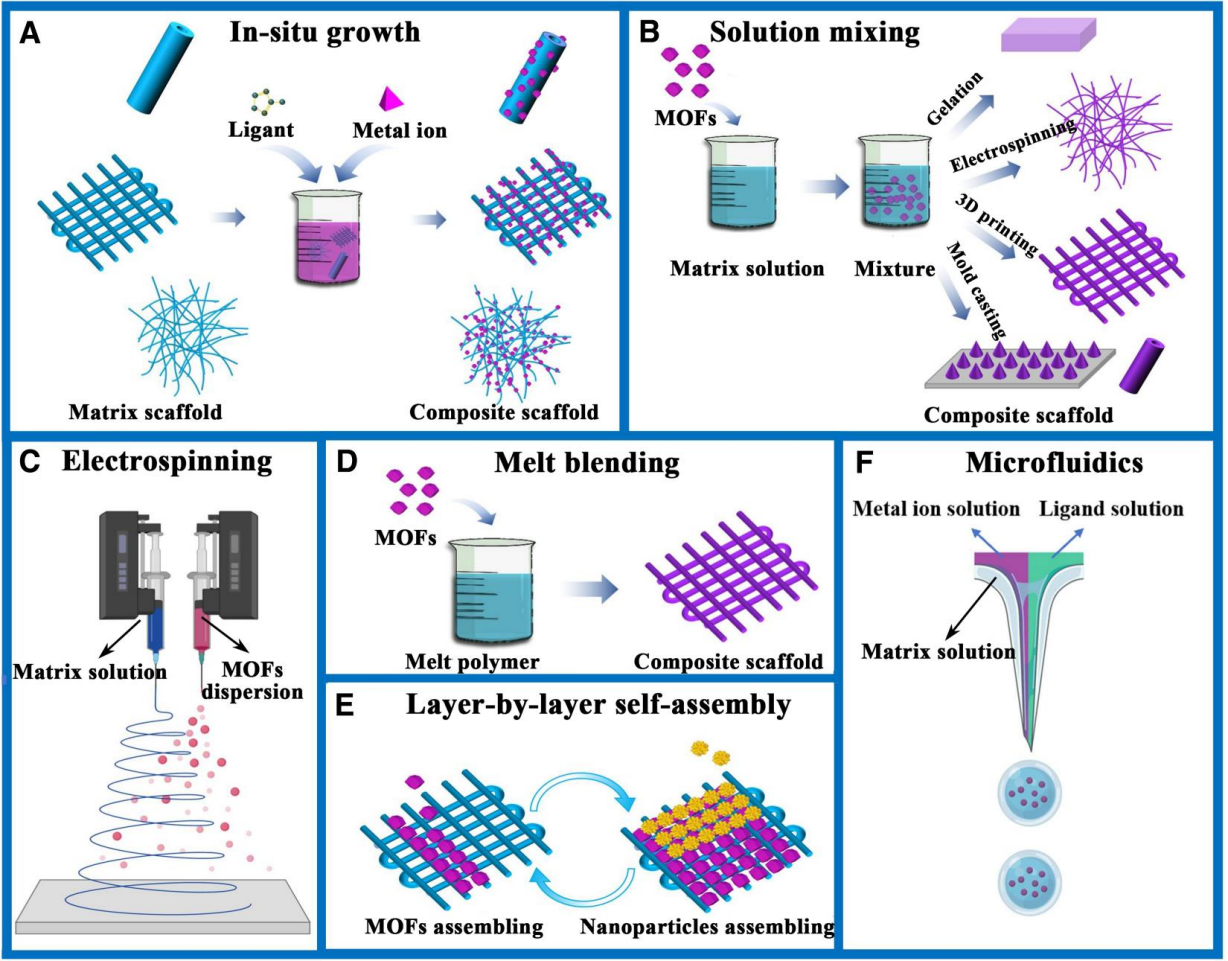
Schematic diagram of the different construction methods of MOFs based composites for tissue engineering. (**A**) In-situ growth method, (**B**) solution mixing method, (**C**) electrospinning method, (**D**) melt blending method, (**E**) layer-by-layer self-assembly method and (**F**) microfluidics method.

The *in situ* growth method refers to growing MOFs crystals directly on the surface of the matrix materials after the basic construction of the scaffolds is completed (Figure 1A). Collagen [58], silk fibroin (SF) [59, 60], gelatin [61], chitosan (CS) [62], hyaluronic acid (HA) [63], β-tricalcium phosphate (β-TCP) [64] and other materials contain numerous active groups, including amino groups, carboxyl groups and phosphate groups, which have been used to load MOFs *in situ* for application in tissue engineering or biomedicine. In the absence of active groups, although a nano-MOFs structure covering the surface of the matrix can be formed, there is no chemical bond interaction between the particles and the matrix, and the connection is not strong, resulting in obvious separation of the particles from the matrix. Therefore, in some studies, the surface of inert materials was modified to enhance the interaction between MOFs particles and the matrix material. For example, thermal oxidation treatment [36] and polydopamine (PDA) coating [52] have been applied to modify Ti-based materials for the *in situ* loading of MOFs. Collagen [65] and gelatin [61] were blended with polycaprolactone (PCL) to promote the *in situ* loading of MOFs. The advantage of the *in situ* growth method is that the MOFs particles can be directly exposed on the surface of the substrate material and can produce a very strong direct effect on the target cells or tissues. However, this method requires active groups on the surface of the substrate material to serve as active sites for nanoparticle growth.

### Solution mixing

In solution mixing or solution blending, MOFs particles and matrix materials are mixed in solution, and the nanoparticles are evenly dispersed in solution by stirring or ultrasonic dispersion. The blended solution can be used for the direct preparation of hydrogels [63, 66], electrospinning fibers [38, 67–69], 3D printing scaffolds [70–72] or mold casting scaffolds [73, 74] (Figure 1B).

In this method, MOFs nanoparticles need to be synthesized first, and there is no need to produce strong chemical bonds between nanoparticles and the matrix as in the *in situ* growth method. Moreover, the solution blending method has low requirements for matrix materials, simple operation and a wide application range. Therefore, this method is currently the most widely used for the preparation of MOFs-based functional biomaterials.

### Electrospinning

Electrospinning technology refers to the application of high-voltage power to flow or deform a charged polymer solution or to melt the material in an electrostatic field, followed by solvent evaporation or melt cooling and solidification to obtain a fibrous material. Micron-sized or nanofiber networks constructed by electrospinning can closely mimic the structure of the extracellular matrix and have therefore been widely used in tissue engineering [75–77].

Although in the above-mentioned solution blending methods, the blended solutions can be used for electrospinning, electrospinning is only a forming method and is not a direct recombination method. Electrospinning technology can be directly used to form composites of MOFs particles and matrix materials. In some studies, double needles were used to electrospin pure poly(lactic-co-glycolic acid) (PLGA) fibers with one needle and spray MOFs nanoparticle dispersions with the other needle (Figure 1C). The MOFs nanoparticles can be evenly dispersed on the fiber surface [78].

### Melt blending

Melt blending is a method of preparing a polymer/MOFs blend solution by mixing polymer components with MOFs particles in a mixing device at a temperature above the polymer viscosity temperature and then cooling and molding the mixture to prepare the corresponding polymer/MOFs composite scaffolds (Figure 1D). The basic requirements of this method are that the polymer should be a thermoplastic polymer and that the melting temperature should be lower than the decomposition temperature of MOFs. Therefore, MOFs materials with high thermal stability, such as zeolitic imidazolate framework-8 (ZIF-8), are more suitable for this method [71, 79]. ZIF-8 nanoparticles were introduced into the poly-l-lactic acid (PLLA) matrix, and then a composite scaffold for bone defect repair was constructed by laser burning technology [79]. This method is essentially a melt blending method to form a composite of the two materials. ZIF-8 nanoparticles were added to molten PCL, and then 3D printing was used to successfully construct a ZIF-8-modified PCL bone repair scaffold [71]. Recently, a silver-/silver-chloride-decorated iron-based MOF (NH_2_-MIL-88B(Fe)) was introduced into PCL by melt electrowriting to construct highly ordered scaffolds with antibacterial properties and magnetic resonance imaging (MRI) visualization ability [80]. These multifunctional scaffolds have great potential for tissue engineering because they reduce the risk of postoperative infections and enable noninvasive monitoring after implantation.

Compared with the three methods mentioned above, the melt blending method is more suitable for commercial use because it is environmentally friendly, without the use of organic solvents, and is suitable for larger-scale processes. However, in the field of tissue engineering, bioactive factors are easily inactivated at high temperature, and bioactive materials, especially protein-based biomaterials, are easily denatured. Therefore, the melt-mixing method is not suitable for the direct preparation of bioactive scaffolds. The authors believe that combining melt mixing with other methods may be a research direction for commercial MOFs functionalized scaffolds.

### Other methods

In addition to the several mainstream preparation methods mentioned above, some other preparation methods have been applied to fabricate MOFs functionalized tissue engineering scaffolds. For example, a layer-by-layer self-assembly technique was used to load MOFs with environmentally responsive properties on the surface of gelatin scaffolds prepared by 3D printing [81] (Figure 1E). A coaxial capillary microfluidic spinning technique was used to fabricate vitamin-based core-shell microfibers [82]. A microfluidic electrospray approach was used to synthesize niacin MOFs encapsulated microcapsules *in situ* for wound healing [83] (Figure 1F). We believe that with the rapid development of materials science, an increasing number of advanced technologies will be applied to the construction of MOFs functionalized composites for tissue engineering.

## Function of MOFs in MOFs-based biomaterials for tissue engineering

### Drug/bioactive factor loading

Structurally, at the molecular scale, the frameworks in MOFs molecules are characterized by large spaces and small windows, enabling them to encapsulate small molecules [84, 85]. At the nano/microscale, MOFs contain numerous open, semiopen and closed nanopores, mesopores or micropores, allowing them to effectively load and protect bioactive factors or drugs and achieve long-term stable release of the loaded molecules [31, 86]. The pore size and morphology of MOFs can be tuned according to the type of drug to be loaded and the purpose of drug delivery [54]. It is important to consider the pore size of the chosen MOFs when designing a drug release system because the pore size of the MOFs directly affects the loading efficiency, release rate and release selectivity of the loaded drug [30, 31]. For instance, the larger pore size (4–29 Å) of the Materials of Institute Lavoisier (MIL)-n family greatly increased its drug loading rate (13.6–94.3%) compared to that of the ZIF-n family [87]. In general, drugs can be loaded into MOFs via three approaches—*in situ* encapsulation, pore encapsulation and surface modification (including modification through intermolecular forces or covalent linkages) [87, 88]. In addition, the direct use of small molecules with therapeutic effects as organic ligands in MOFs synthesis is another a promising approach for drug loading [47, 50]. In terms of environmental responsiveness, some MOFs have the ability to selectively undergo collapse or degradation to release encapsulants in response to changes in the body’s internal environment (e.g. changes in the microenvironment due to acute injury or cancer) or to external stimuli (e.g. pH, magnetic, ionic, temperature, pressure, light or humidity stimuli) [89–93]. This ability allows the spatial and temporal control of drug release, ultimately achieving targeted drug delivery. In addition, several studies have demonstrated the potential of MOFs to penetrate the blood–brain barrier [94–96]. Therefore, MOFs have attracted great interest in the field of drug or bioactive factor encapsulation.

One of the aims of tissue engineering is to repair damaged tissues or organs. During the repair process, it is important to deliver drugs with anti-inflammatory, antibacterial or tissue repair-promoting functions. Combining MOFs containing relevant drugs with tissue-engineered scaffold materials can provide physical support along with long-term stable drug therapy and greatly improve the repair efficiency of damaged tissues [52, 97, 98]. For instance, methyl vanillin-loaded MOFs can enhance the osteogenic differentiation of PDA-modified titanium-based bone repair implants [52]. The ZIF-8 nanodelivery system loaded with simvastatin promotes osteogenesis while inhibiting lipogenesis [98]. Antibiotics loaded with MOFs (e.g. vancomycin [50], curcumin [51] and levofloxacin [99]) have strong anti-infective capabilities in wound healing or infected bone repair. In addition to conventional drugs, MOFs can also encapsulate some gene therapy drugs, such as microRNAs (miRNAs), for tissue engineering. Feng *et al.* [100] attempted to load pro-angiogenic miR-21 and pro-osteogenic miR-5106 in ZIF-8 nanoparticles using a one-pot method. *In vitro* studies showed that miR@ZIF-8 nanoparticles have high miRNA loading efficiency. In addition, since ZIF-8 encapsulates miRNA in its pores, it protects miRNA from RNase degradation, ultimately providing higher miRNA transfection efficiency and more stable cellular expression than the commercially available Lipo3000. In addition, because of the pH responsiveness of ZIF-8, the composite nanoparticles could be disassembled in acidic endolysosomes, thus triggering miRNA release in the cell. *In vitro* studies showed that ZIF-8 nanoparticles loaded with two different functional miRNAs effectively promoted both angiogenesis and the osteogenic differentiation of bone marrow mesenchymal stem cells. Next, miR@ZIF-8 nanoparticles were introduced into alginate gel to prepare a composite hydrogel, which was then used for cranial defect repair. The results showed that the composite hydrogel could effectively promote angiogenesis and bone formation. This study presents new ideas for using MOFs as an efficient therapeutic nucleic acid delivery system for tissue regeneration.

Bioactive factors are critical in promoting regeneration and building microenvironments [101–103]. In constructing tissue-engineered scaffolds, loading factors with specific functions on the surface or inside of the scaffolds by coating, direct adsorption and grafting is an effective means of improving cell adhesion, promoting regeneration and facilitating vascularization of tissue-engineered scaffolds [104–107]. However, most of the factors are proteins or peptides, and the factors loaded in the tissue engineering scaffolds are easily inactivated without good protection, resulting in the inability of the tissue engineering scaffolds to maintain the appropriate function for a long time. The overly short action time limits the repair effect of tissue-engineered scaffolds. MOFs can load bioactive factors and maintain their bioactivity. Basic fibroblast growth factor [108], bone morphogenetic proteins [81, 109] and the cytokine interleukin 4 [110] have been successfully loaded in MOFs and can maintain long-term stable activity in tissue repair, ultimately promoting tissue regeneration.

To date, drug delivery is the most widely and deeply studied function of MOFs. We believe that drug delivery is also the most important role of MOFs in future clinical applications. MOFs themselves also have anti-inflammatory, antimicrobial, vasodilatory or regenerative effects. Therefore, in the following descriptions of the specific functions of MOFs in MOFs-based composite scaffolds, unless otherwise stated, the functions refer to the effects of the MOFs themselves rather than the effects of the loaded corresponding drugs.

### Antibacterial

**Figure 2.**
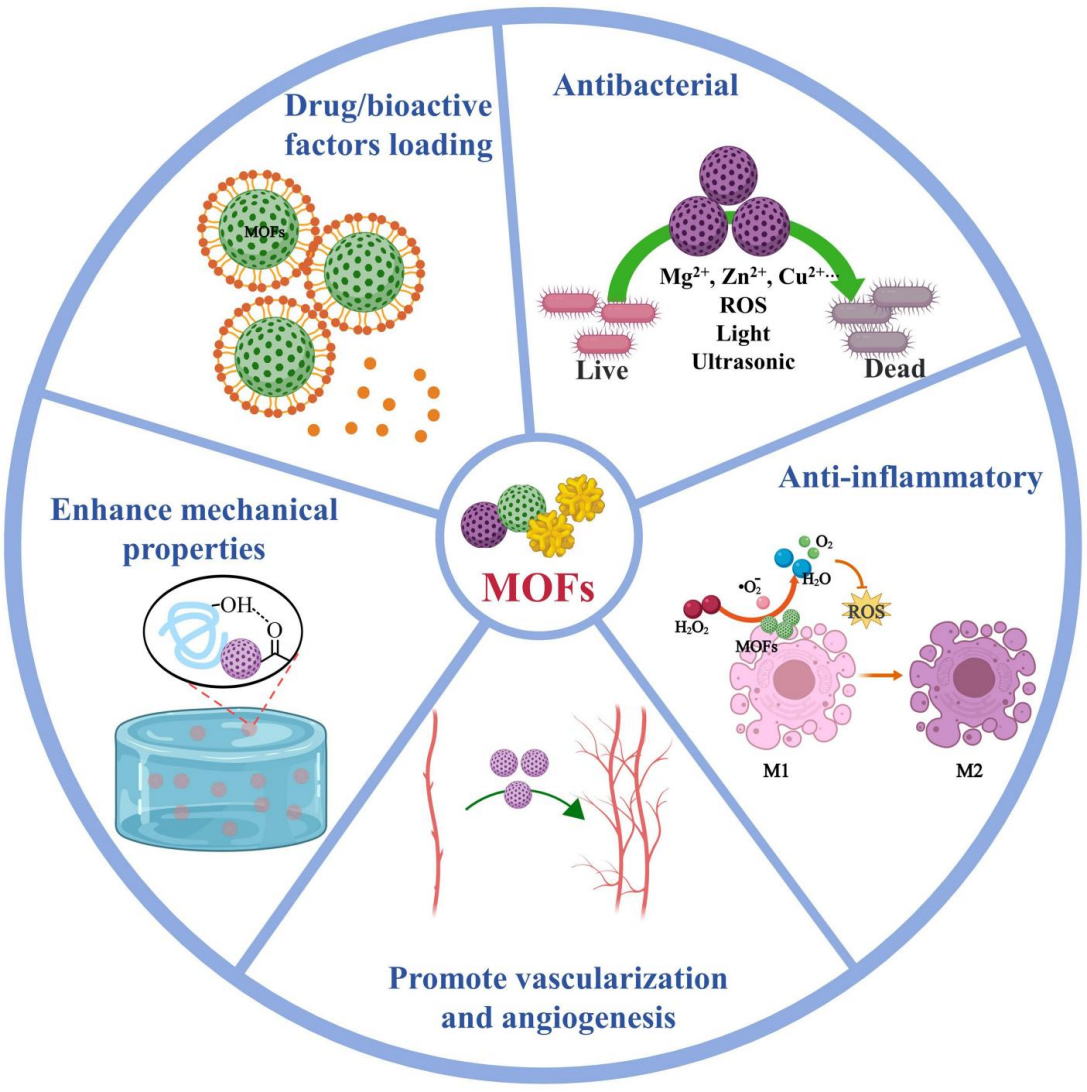
Schematic diagram of the function of MOFs in tissue engineering.

MOFs nanoparticles are often used directly for antimicrobial therapy or combined with other materials to enhance the antimicrobial function of the material (Figure 2). In addition to the loading of antimicrobial drugs, the antimicrobial mechanisms of MOFs mainly include the following aspects. First, some MOFs contain antimicrobial metal ions (such as Zn^2+^, Cu^2+^ and Zr^2+^) [111–115]. Under certain conditions, with the gradual decomposition of MOFs, the continuous release of metal ions can be effective antibacterial agents. For example, Si *et al*. coated the surface of PDA-modified titanium with methyl vanillate-modified ZIF-8 (MV@ZIF-8) to improve the clinical applicability of titanium-based materials and to enhance the anti-infection and bone regeneration ability [52]. A systematic study of the antibacterial mechanism of MOFs revealed that ZIF-8 can release Zn^2+^ slowly and continuously. Zn^2+^ attaches to negatively charged membranes through electrostatic interactions. Zn^2+^ adsorption on cell membranes may increase the permeability of cell membranes, and MV@ZIF-8 nanoparticles are able to form pores in cell membranes, disrupting the integrity of the membranes and thus killing bacteria. In addition, Zn^2+^ promoted the production of large amounts of reactive oxygen species (ROS) in bacteria, which can induce oxidative stress and damage DNA and RNA, thereby disrupting protein expression and thus causing extensive oxidative damage to bacteria. In addition to releasing Zn^2+^, the Zn nodes in ZIF-8 can also be used as reaction sites to effectively kill bacteria.

Second, photosensitive MOFs can absorb energy at appropriate wavelengths of light and transfer the energy to the surrounding oxygen to generate ROS, which produces significant cytotoxicity and thus kills bacteria. Photosensitizing MOFs can kill bacteria by absorbing energy at appropriate wavelengths under certain wavelengths of light and transferring the energy to the surrounding oxygen, thereby generating ROS and producing significant toxicity [116, 117]. Zhang *et al*. [116] first synthesized a photosensitive antimicrobial MOF, PCN-224, using 5,10,15,20-tetrakis(4-methoxycarbonylphenyl)porphyrin as the ligand and Zr_6_ clusters as metal nodes. PCN-224 was recombined with Ag nanoparticles and coated with sodium hyaluronate for the treatment of wound infections. PCN-224 produces ROS under visible light irradiation, which synergistically interact with silver nanoparticles to efficiently kill bacteria. Since then, some researchers have prepared bimetallic PCN-224 (Zr/Ti) by combining nanoparticles PCN-224 with Ti through a cation exchange strategy and replacing part of Zr with Ti [78]. Compared with PCN-224, PCN-224 (Zr/Ti) had significantly higher the photocatalytic performance for ROS generation under visible light.

The third antibacterial mechanism of MOFs is that some MOFs with photothermal properties can kill bacteria by local overheating under certain light radiation. Prussian blue nanoparticles (PBNPs) are typical MOFs with photothermal properties [118–120]. The composite hydrogel prepared by blending PBNPs with modified CS showed excellent photothermal properties under 808 nm near infrared (NIR) light irradiation [118]. The hydrogel can capture bacteria by electrostatic adsorption, and the subsequent localized heat generated by the PBNPs under near-infrared irradiation kills large numbers of adsorbed bacteria. Notably, the application of MOFs based on photodynamic and photothermal mechanisms is limited by the tissue penetration of light, and they are more suitable for the treatment of open superficial wound repair. A more comprehensive and detailed summary of MOFs and their composites for skin wound phototherapy is available from Yang *et al*. [56] and will not be presented more specifically here. The application of MOFs to the regeneration of infected tissues based on their antimicrobial properties is not limited to a specific antimicrobial mechanism. Many studies have focused on integrating multiple antimicrobial mechanisms to achieve better antimicrobial effects. Silver nanoparticles were used to modify MOFs to improve the photodynamic therapy efficiency [121]. The constructed composite nanoparticle system exhibited excellent synergistic bactericidal effects through various mechanisms, including disruption of the cell membrane and interference with the translation process.

In addition to the antibacterial mechanisms mentioned above, sonodynamic therapy synergistic sterilization is also an important approach to sterilization using MOFs [122–124]. The application of ultrasound-responsive antimicrobial MOFs-based composite scaffolds to repair defective tissues has not yet been reported but would be a promising research direction due to the deep tissue penetration and noninvasiveness of SDT.

Due to their excellent antibacterial properties, MOFs have been widely used in wound treatment and bone repair, as both applications are prone to infection. MOFs with antimicrobial properties create a microenvironment that is not conducive to bacterial survival, such as excess ROS and localized hyperthermia. This microenvironment may also cause damage to normal tissues and cells. Therefore, more attention should be given to the safety of MOFs and the controllability of their effects on the microenvironment in practical applications.

### Promote vascularization and angiogenesis

In tissue engineering research, regardless of the type of tissue, the implant (artificial or autologous) requires an adequate blood supply to obtain nutrients and oxygen and to remove metabolic wastes [125, 126]. In the current process of tissue repair with biomaterials, the vast majority of tissue regeneration occurs in the peripheral part of the scaffold, while tissue regeneration in the central area is usually poor and cannot achieve complete healing. The main reason for this is that newly formed tissues lack a blood system to supply them with blood oxygen, which eventually leads to cell death and tissue degeneration. Therefore, larger tissues require the rapid formation of new capillary networks, where new blood vessels sprout from host vessels and interconnect by penetrating the extracellular matrix. Therefore, biocompatible materials that promote blood vessel formation are very important in tissue engineering research. Previous studies have shown that the presence of MOFs can promote vascularization and angiogenesis [127–131]. Researchers mostly believe that MOFs promote angiogenesis mainly due to the metal ions produced by their decomposition. For example, magnesium stimulates cell migration and proliferation, thereby promoting angiogenesis and wound healing [132]. Based on this, Yin *et al*. [133] introduced Mg-based MOFs (Mg-MOFs) into a poly(γ-glutamic acid) (γ-PGA) hydrogel and then loaded them in a microneedle (MN) patch. The Mg-MOFs could promote angiogenesis by slowly releasing Mg^2+^. *In vitro* tube formation studies showed that Mg-MOFs could significantly increase the tubule formation process of human umbilical vein endothelial cells (HUVECs), as reflected by the significant increase in branch point and average tube length. The results of the *in vivo* study showed that the addition of Mg-MOFs could significantly increase the capillary density of the wound, indicating that this treatment could improve the rate of neovascularization. Since ZIF-8 can release Zn^2+^, which plays a positive role in angiogenesis, Liu *et al*. introduced it into catechol-chitosan (CA-CS) to construct a composite hydrogel (CA-CS/Z) [134]. The *in vivo* and *in vitro* results showed that ZIF-8 could significantly enhance the ability of hydrogels to promote angiogenesis. To further understand the mechanism by which CA-CS/Z composite hydrogels promote angiogenesis, vascular endothelial growth factor (Vegf-a) secretion and gene molecular level analysis were performed. Studies have shown that in the presence of ZIF-8 nanoparticles, the expression levels of the Vegf-a gene and eNos gene in rat bone marrow mesenchymal stem cells were significantly increased, leading to enhanced vascularization. However, there are few studies elucidating the relationship between sustained release of metal ions and angiogenesis.

### Enhance mechanical properties

Many natural biomaterials, such as SF, collagen and HA, are limited by poor mechanical properties for the construction of tissue engineering scaffolds [135–137]. The addition of nanoparticles is a very simple and effective way to improve mechanical properties. MOFs nanoparticles stand out among a variety of nanoparticle candidates since their unique structure gives them extraordinary mechanical properties and chemical interactions. In PLLA/ZIF-8 composites, ZIF-8 particles and the PLLA matrix can form strong interfacial bonds, which significantly improve the tensile strength, compressive strength and hardness of PLLA [79]. In addition, it has been reported that the addition of ZIF-8 can significantly improve the mechanical properties of HA [63]. The addition of ZIF-8 nanoparticles could effectively regulate and enhance the mechanical properties of bioprinted hydrogel constructs [138]. Han *et al*. added PBNPs to quaternary ammonium and double-bond-modified CS to synthesize photosensitive hydrogels. The PBNPs could strengthen the mechanical properties of the hydrogel as a crosslinker. It has been shown that MOFs can produce synergistic interactions with SF at the secondary structure level, thereby enhancing the mechanical strength of SF [139]. The enhancement and modulation of hydrogels by MOFs provide new ideas for the development of more stable 3D printing bioinks with suitable mechanical properties.

### Anti-inflammatory

The inflammatory response is an important factor affecting the repair and regeneration of damaged tissues [140, 141]. It is generally believed that persistent inflammation has a negative effect on tissue regeneration [142–144]. Therefore, timely relief of inflammation is essential for the regeneration of injured tissues. Many studies have shown that MOFs are promising anti-inflammatory materials [145–147]. For instance, Kang *et al*. introduced Mg-based MOFs (Mg-GA) with gallic acid (GA) into PLGA as organic ligands to achieve anti-inflammatory effects in the process of bone injury repair. Mg^2+^ plays an important role in the regulation of the inflammatory response [148, 149]. In addition, GA, a polyphenolic compound with antioxidant and anti-inflammatory effects, can attenuate the LPS-induced expression of inflammatory mediators and the production of ROS in RAW264.7 macrophages [150]. The *in vivo* results further confirmed the anti-inflammatory effect of Mg-GA addition. Regarding the anti-inflammatory mechanism, the group speculated that GA attenuated the inflammatory response by reducing the release of inflammatory cytokines, chemokines, adhesion molecules and inflammatory cell infiltration, based on previous findings [151]. However, this mechanism was not confirmed by specific evidence in this study. Notably, this study also functionalized Mg-GA with exosomes and loaded it into the scaffold to further improve the anti-inflammatory, osteogenic and vascularization abilities of the scaffold, thereby accelerating bone regeneration. This work is extremely creative and provides valuable new insights into the design of exosome-functionalized nanocomposite scaffolds with accelerated bone regeneration. In our opinion, there is great value for further research on this topic. The mechanisms of the anti-inflammatory, osteogenic and vascularization-promoting effects of Mg-GA and EXO-Mg-GA can be further studied in detail.

According to current reports, the anti-inflammatory mechanism of MOFs is mainly to reduce oxidative stress by scavenging ROS due to their high superoxide dismutase-like (SOD-like) and catalase-like (CAT-like) activities [64, 120, 152]. For example, Shu *et al*. [153] suggested that Zn-based MOFs are a promising material for establishing an anti-inflammatory microenvironment because Zn is an essential element with anti-inflammatory properties by regulating free radical formation in the human body. Thus, they designed a bioceramic scaffold to repair osteochondral defects by functionalizing β-TCP and a zinc-cobalt bimetallic organic framework (Zn/Co-MOF). The results showed that the expression of inflammatory factors was inhibited alongside the reduction in ROS levels, suggesting that Zn/Co-MOFs can help to construct an anti-inflammatory microenvironment, as expected. However, as we mentioned above, the researchers mentioned in the antibacterial mechanism of MOFs that MOFs can increase the level of ROS in bacteria to promote oxidative stress and kill bacteria [52]. In this study, MOFs obviously reduced the level of ROS and thereby reduced oxidative stress. What are the causes and underlying mechanisms of these opposite research results? Does the regulation of ROS levels by MOFs depend on the number, type or duration of action of MOFs? Furthermore, does the type of object (bacteria or cells) matter? What is the underlying mechanism when the same MOFs have both bactericidal and anti-inflammatory effects? Do MOFs promote or inhibit ROS levels? To the best of my knowledge, there are few relevant studies. Research on the function of MOFs in the field of tissue engineering is still incomplete, and more detailed and in-depth researches are necessary.

In addition, the relationship between inflammation and regeneration is complex. Inflammation is often referred to as the “double-edged sword” in tissue regeneration [154]. At present, the focus of related research is the use of MOFs for simple anti-inflammatory effects. Neither selective anti-inflammation according to the developmental stage of inflammation during injury nor anti-inflammatory mechanisms have been studied in more depth. We propose that the excellent responsiveness of MOFs to environmental stimuli can be exploited more flexibly to achieve more precise (e.g. temporal and spatial) regulation of inflammation.

## Application of MOFs in tissue engineering

### Bone/cartilage

**Figure 3.**
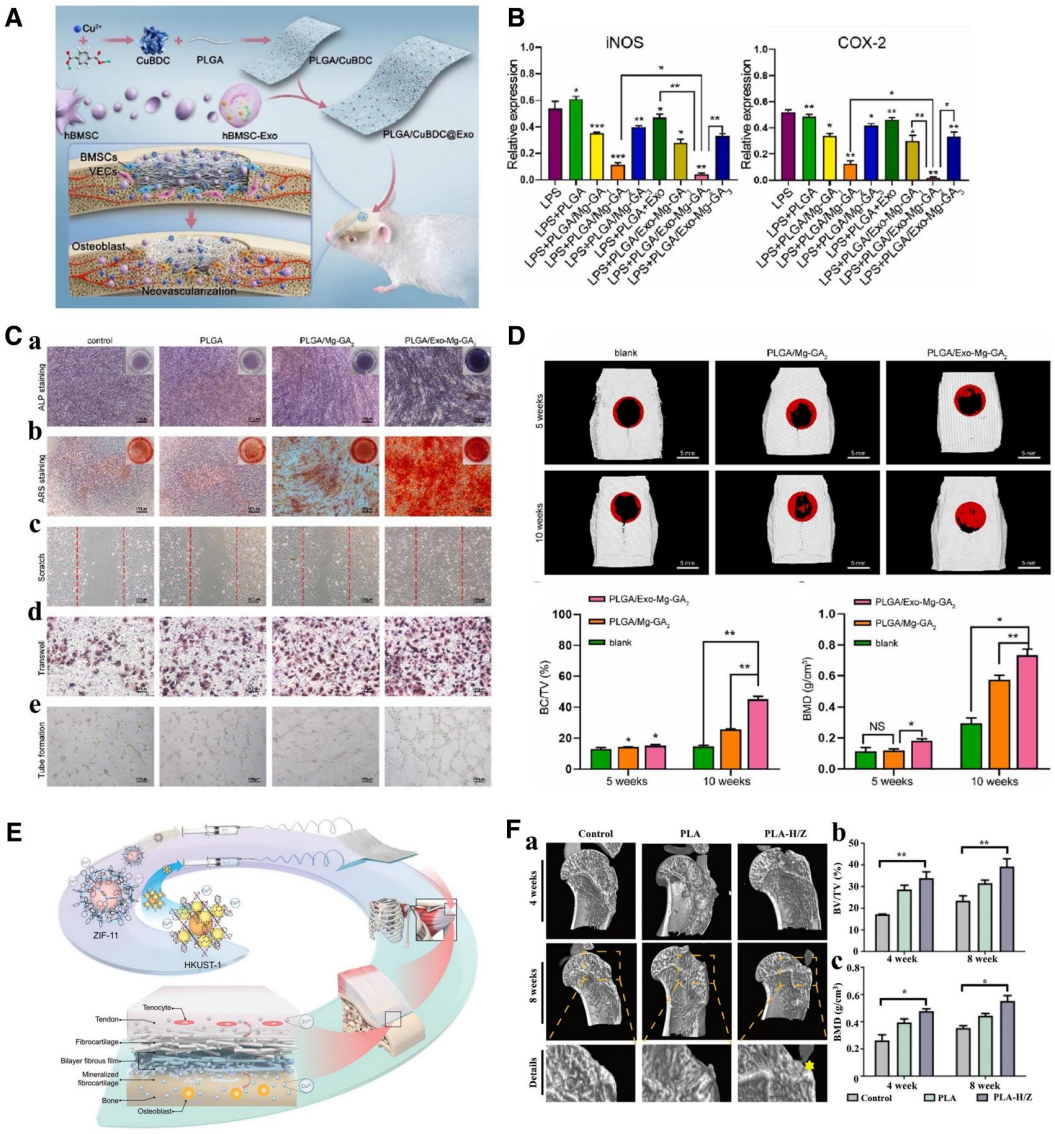
(**A**) Schematic diagram of the exosome-laden PLGA/CuBDC scaffold for the repair of bone defects. Reproduced from Ref. [128] with permission of Elsevier, © 2023. (**B**) Expression of iNOS and COX-2 in RAW264.7 cells cultured on MOFs and exosome-functionalized PLGA scaffolds. (**C**) *In vitro* osteogenic and angiogenic effects of extracts from different scaffolds. (**D**) Bone regeneration capability of the exosome-coated Mg-GA MOF scaffolds. (**B–D**) Reproduced from Ref. [38]. This article is licensed under a Creative Commons Attribution 4.0 International License. (**E**) Schematic diagram of the fabrication and implantation of the MOFs functionalized nanofibrous membrane. (**F**) Osteogenesis evaluation of the membranes *in vivo*. (**E** and **F**) Reproduced from Ref. [164] with permission of John Wiley & Sons, © 2022. (*P < 0.05, **P < 0.01, ***P < 0.001)

The repair and treatment of bone defects have always been a great clinical challenge [155–157]. For traumatic bone injury, the first physiological response at the site of injury is inflammation, and at this stage, a hematoma is formed. Subsequently, the hematoma becomes a cartilaginous callus, and bone repair enters the ossification stage, accompanied by hardening and strengthening of the cartilage callus. Finally, with the combined action of osteoblasts and osteoclasts at the site of injury, bone remodeling is complete, and the callus is completely absorbed. Therefore, bone tissue repair is a long-term and highly complex process involving the process of inflammation, osteogenesis, angiogenesis and so on. Many synthetic or natural biomaterials, such as collagen, PCL, PLLA and PLGA, have been applied in bone tissue engineering [158–163]. However, although these materials have good biocompatibility and present their own advantages in practical applications, their low osteogenic induction ability, insufficient vascularization and lack of anti-inflammatory effects ultimately lead to poor bone injury repair. MOFs have been widely studied in the field of bone regeneration due to their proangiogenic, anti-inflammatory and drug loading functions [52, 67, 164, 165]. ZIF-8-modified multifunctional hydrogels were designed to stabilize the bone graft environment, ensure blood supply, promote osteogenic differentiation and accelerate bone remodeling [134]. The effects of sustained Ca^2+^ and Sr^2+^ release by MOFs on the osteogenic activity of human cells was investigated [166]. CaSr-MOFs themselves can upregulate osteogenic genes in human mesenchymal stem cells. Functionalized composite scaffolds were constructed by co-fixing MOFs with exosomes on PLGA porous polymer fibers (Figure 3A) [38, 128]. The *in vitro* results demonstrated that the addition of Mg-GA could significantly reduce the expression of iNOS and COX-2 in RAW264.7 cells grown on PLGA scaffolds (Figure 3B). The dual synergistic release of bioactive copper ions and exosomes from the composite scaffold can promote osteogenesis and angiogenesis, thereby achieving acellular bone regeneration (Figure 3C and D) [38]. Bipolar metal-flexible electrospun fiber membranes based on two MOFs (ZIF-11 and HKUST-1) were fabricated for gradient healing in tendon–bone interface regeneration (Figure 3E and F) [164]. Mild hyperthermia has positive effects on bone repair [167]. Therefore, ZIF-8-PDA nanoparticles with photothermal conversion properties were introduced into scaffolds for the repair of skull defects [168]. In addition, a variety of drugs (e.g. alendronate [169], dexamethasone [170] and simvastatin [98]) used to treat bone damage repair were loaded into MOFs to achieve more sustained and more effective release, thus further promoting bone repair.

The inflammatory microenvironment of osteoarthritis-induced osteochondral defects is closely related to the excessive production of ROS. Excessive ROS-induced oxidative stress leads to the aggravation of osteochondral defects and the difficulty of osteochondral tissue regeneration. Therefore, the construction of bone tissue engineering scaffolds with the ability to clear ROS and promote bone regeneration is very important for the repair of osteochondral defects induced by osteoarthritis. A Zn/Co-MOF-functionalized bioceramic scaffold was designed and fabricated for the repair of osteochondral defects in the OA environment [64]. This study demonstrated that MOFs endow scaffolds with excellent antioxidant and anti-inflammatory properties, protect cells from ROS and reduce the oxidative stress response, and MOF-TCP scaffolds accelerated the global regeneration of cartilage and subchondral bone in severe osteochondral defects. In particular, the effect of the loaded MOFs content was studied in great detail. The content of the MOFs loaded on the scaffold surface *in situ* was controlled by adjusting the concentration of the reaction solution, and the optimal preparation concentration was selected. This has a very good reference value for future research. Apart from MOFs themselves, derivatives of MOFs have also been applied to promote bone regeneration after injury. MOF-derived CuO@ZnO particles were loaded onto PDA-modified titanium alloy to achieve vascularized bone regeneration [43].

Currently, MOFs have been widely used in bone tissue repair. In terms of the types of MOFs studied, compared with the large family of MOFs, the types of MOFs that have been applied to bone tissue engineering repair are very limited, mostly focusing on the study of ZIF-8-based composites. The research on other kinds of MOFs has yet to be expanded. As for the depth of research, the synergistic effect between the components of MOFs-based scaffold materials, the interaction mechanism between scaffold materials and the microenvironment and the intrinsic mechanism of MOFs for repairing bone defects have to be further explored.

### Skin

**Figure 4.**
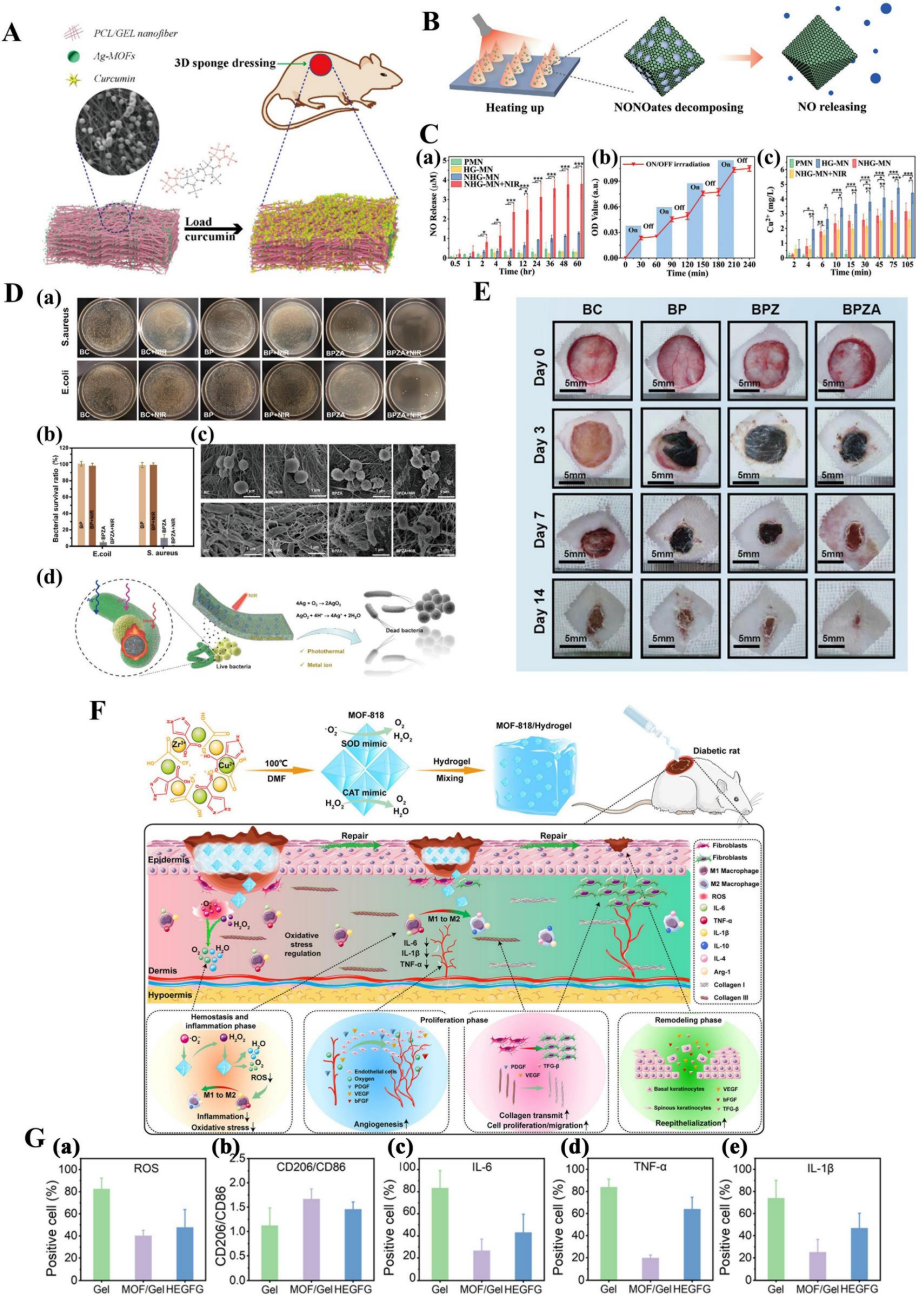
(**A**) Schematic diagram of the MOFs-functionalized 3D fibrous sponge. Reproduced from Ref. [61] with permission of Elsevier, © 2022. (**B**) Schematic diagram of the photothermal-responsive NO delivery process. (**C**) Photothermal-responsive NO delivery of porous MOFs MN patches. (**B** and **C**) Reproduced from Ref. [180]. This article is licensed under a Creative Commons Attribution 4.0 International License. (**D**) Antibacterial activity of the BC/PDA/ZIF-8/Ag hydrogel. (**E**) Representative pictures of full-thickness skin defects in rat models. (**D** and **E**) Reproduced from Ref. [127] with permission of Elsevier, © 2023. (**F**) Schematic diagram of the preparation of the MOFs functionalized gel and its mechanisms in diabetic chronic wound healing. (**G**) (a) ROS levels in the wound tissues on day 8, (b) quantification of CD86- and CD206-positive cells, (c–e) quantification of IL-6-, IL-1β- and TNF-α-positive cells. (**G** and **F**) Reproduced from Ref. [152] with permission of American Chemical Society, © 2022.

The skin is the largest organ of the human body and has functions including protection, excretion, the regulation of body temperature and the sensing external stimulation [171]. However, surgery, trauma, burns and physiological abnormalities can lead to severe wounds in a range of skin tissues. Wound healing is one of the most challenging problems in regenerative medicine [172–175]. Wounds can generally be classified into acute and chronic types, and most wounds heal well. However, for patients with physiological diseases such as diabetes and for people with mobility disorders, the wound healing process can be prolonged. Long-term direct contact between the wound and the external environment greatly increases the probability of contact with infectious agents, leading to chronic infection and wound nonhealing [176, 177]. Therefore, prevention and treatment of infection during wound healing are essential. The good antibacterial properties of MOFs endow them with great application potential in antibacterial therapy and wound healing. MOFs-based functional materials have been extensively and deeply studied in wound healing [56, 178, 179]. The forms of MOFs-based composite wound dressings or scaffolds mainly include fibrous sponges (Figure 4A and D), MNs (Figure 4B) or hydrogels (Figure 4F), with hydrogels being the most widely used [61, 127, 152, 180]. Using different methods to load various MOFs onto dressings or scaffolds can enhance the antibacterial properties of dressings or scaffolds against different types of bacteria [51, 127, 181, 182]. For instance, the addition of ZIF-8 can effectively improve the antibacterial ability of bacterial cellulose [127]. A series of phototherapeutic PCN-based MOFs have been introduced into various materials, such as polyvinyl alcohol, sodium alginate (Alg), PDA and CS, for the treatment of infected wounds [116, 183–186]. Furthermore, researchers have used MOFs to encapsulate antibacterial drugs and use the synergistic effect of antibacterial drugs and MOFs to kill bacteria more efficiently [139]. The results of studies have shown that composite dressings have excellent antibacterial properties. The excellent performance of multifunctional MOFs-based composites in wound healing will make them a favored candidate for relevant related research.

The ultimate goal of wound treatment is to promote the regeneration of skin tissue and achieve functional reconstruction. Therefore, in addition to being used for antibacterial effects, MOFs can be designed so that their degradation products of MOFs (metal ions and organic ligands) and loaded components (e.g. ascorbic acid [179], NO [178], 2-deoxy-D-ribose [139], curcumin [51], epigallocatechin gallate [97] and vancomycin [50]) can physiologically induce wound healing. Based on the complexity of the wound healing process, multifunctional MOFs-based composites have been fabricated [50, 133, 187, 188]. As shown in Figure 4B and C, NO-loaded HKUST-1 was encapsulated with graphene oxide to fabricate photothermally responsive composite microparticles [180]. The introduction of the composite microparticles into the MN array patch could promote vascularization, tissue regeneration, and collagen deposition in wound healing. Chao *et al*. [152] established an effective antioxidant system for wound healing by combining MOF-818, which has antioxidant enzyme-like activity, with a hydrogel. This system regulates the microenvironment of chronic wounds by continuously removing ROS, leading to a natural transition from the inflammatory phase to the proliferative phase (Figure 4F and G). This work demonstrated the successful application of antioxidative MOF nanozymes in diabetic wound healing. Yang *et al*. [187] constructed a three-in-one synergistic antibacterial platform based on gallium-based MOFs-coated hollow TiO_2_ nanoshells (H-TiO_2_−x@MOF) for the simultaneous eradication of methicillin-resistant *Staphylococcus aureus* (MRSA) and *Pseudomonas aeruginosa* (PA). In this system, the incorporation of gallium, which exhibited sustained release under acidic conditions, into ferritin could disrupt bacterial redosion-driven biological processes in bacteria. Moreover, hollow TiO_2_ with abundant oxygen defects can achieve high levels of ROS production under visible/NIR irradiation. Thus, the combination of an ROS generator (H-TiO_2_-X) and an antioxidant enzyme inhibitor (gallium) acted synergistically to effectively eradicate MRSA. Taxifolin (TAX)-loaded cyclodextrin MOFs (TAX@CD-MOFs) were introduced into a PCL matrix by electrospinning to construct multifunctional electrospun fibrous membranes (EFMs) [189]. Treatment with TAX@CD-MOFs resulted in the release of TAX, which has various beneficial properties, such as anti-inflammatory, antimicrobial, antioxidant and angiogenesis-promoting effects, suggesting that multifunctional EFMs have promise as dressings for diabetic wounds. A bio-MOF with curcumin as the ligand, Zn^2+^ as the coordination center and vancomycin loaded and coated with quaternary ammonium chitosan was prepared [50]. Then, it was combined with methacrylic anhydride-modified gelatin and methacrylic anhydride-modified Alg through free radical polymerization and the Schiff base reaction to prepare multifunctional composite hydrogels for wound healing. Composite hydrogels have multiple functions, such as intelligent bacterial capture, rapid antibacterial activity, anti-inflammatory activity, angiogenesis, nerve repair, hyperthermia and tissue adhesion. They have shown a great ability to promote the healing of chronic wounds.

### Nerve

**Figure 5.**
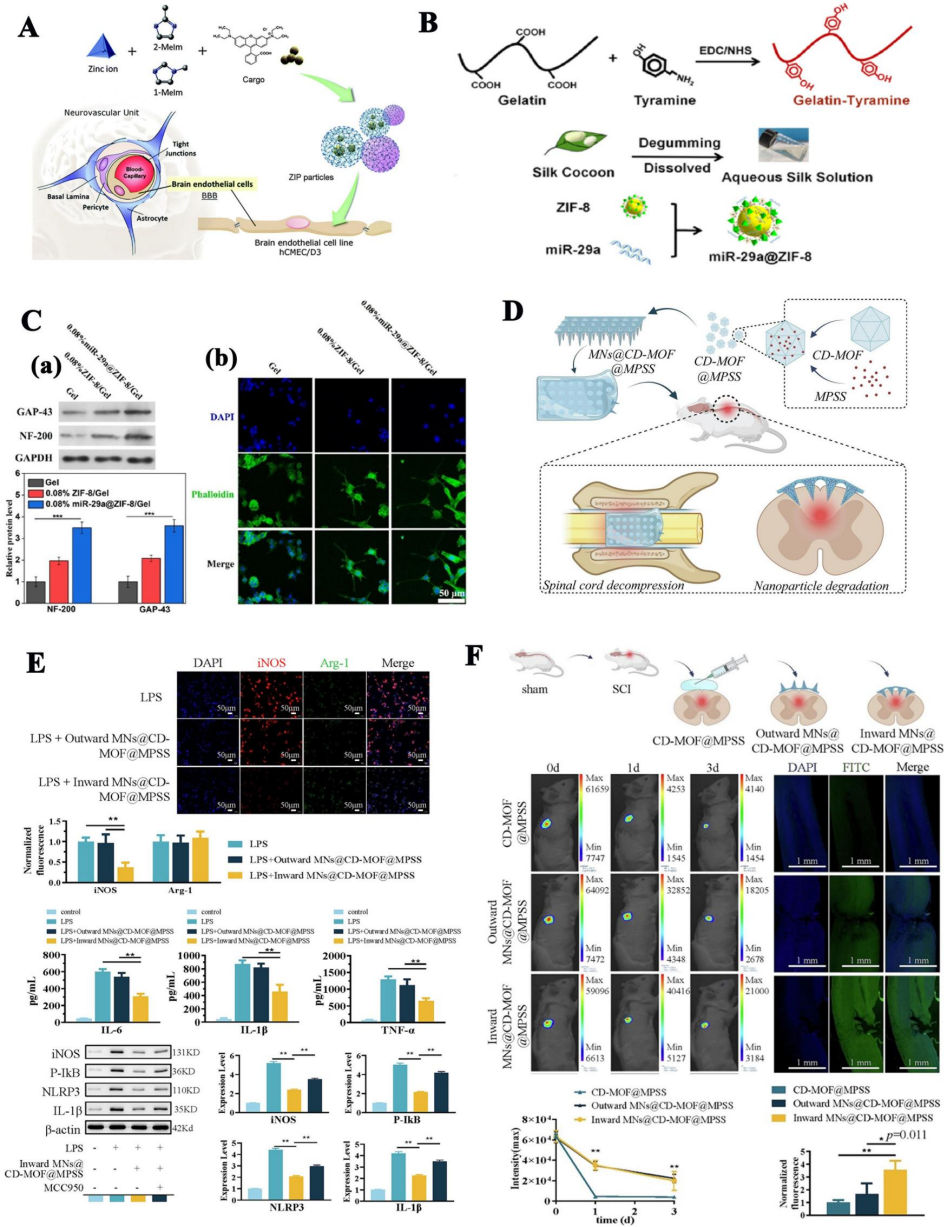
MOFs Functionalized composites applied in nerve tissue engineering. (**A**) Synthesis and assembly of loaded ZIP particles and their uptake into human brain ECs. Reproduced from Ref. [195]. This article is licensed under a Creative Commons Attribution-NonCommercial 3.0 Unported Licence. (**B**) Schematic diagram of the construction of MOFs functionalized conduits and their application in peripheral nerve repair. (**C**) (a) Expression levels of GAP-43 and NF-200 proteins in PC12 cells cultured on different hydrogels (***p < 0.001, One-way ANOVA, Tukey's test). (b) Immunostaining images of PC12 cells cultured on different hydrogels. (**B** and **C**) Reproduced from Ref. [200]. This article is licensed under a Creative Commons Attribution 4.0 International License. (**D**) Schematic diagram of the construction of MOFs functionalized MNs and their application in spinal cord injury. (**E**) MNs@CD-MOF@MPSS reduces LPS-induced M1 by decreasing the NLRP3 inflammasome. (**F**) Validation effects of MNs@CD-MOF@MPSS *in vivo*. (*p < 0.05, and **p < 0.001 versus inward MNs@CD-MOF@MPSS group, n = 5) (**D–F**) Reproduced from Ref. [203] with permission of Elsevier, © 2023.

Nerve injury or defects are common clinical diseases whose poor prognosis and high disability rate seriously affect the quality of life of patients. To repair damaged peripheral or central nerves, a variety of biomaterials have been used to construct tissue-engineered nerves [190–192]. The repair of nerve defects is a very complex process involving the immune response, nerve regeneration, angiogenesis, scar formation and other processes. In the process of constructing tissue-engineered nerves, in addition to meeting basic biocompatibility, nontoxicity and mechanical support requirements, a microenvironment conducive to nerve regeneration should also be formed [193, 194]. Obviously, a single biological material cannot meet the above requirements. Therefore, the preparation of multifunctional composites with special functions (including but not limited to inhibiting inflammation, inhibiting scar formation, promoting vascularization, promoting cell migration and axonal extension, etc.) has attracted much attention in the field of nerve tissue engineering. To date, MOFs-based functional biomaterials have been applied less extensively in the field of neurology than in that of bone and skin, belonging to the primary research stage. Moreover, the application of MOFs in the field of neurology focuses more on the direct effects of MOFs or MOFs-based composite nanoparticles on nerve cells or the damaged peripheral or central nervous system [195–198]. A biodegradable zinc imidazolate polymer (ZIP) showed great potential as a new brain-targeted therapeutic platform because it can cross the brain endothelial cell (EC) membrane (Figure 5A) [195]. Recently, ZIF-8-capped SOD and Fe_3_O_4_ nanoparticles (SFZ-NPs) were developed [196]. Since ZIF-8 can be degraded in an acidic environment, SFZ-NPs can expose more numerous active sites in a weakly acidic inflammatory environment. Furthermore, the cascade of SOD-like and CAT-like enzyme activities enables SFZ-NPs to reduce oxidative stress, modulate the proinflammatory microenvironment and ultimately improve analgesia. Mechanistic analysis showed that SFZ-NPs could effectively inhibit the activation of astrocytes and microglia and reduce neuroinflammatory damage by reversing the phosphorylation process of the MAPK signaling pathway and converting ROS to O_2_. Although this work was applied only to the treatment of pain and did not involve nerve-related tissue engineering research (repair or regeneration), it has implications for possible future related research.

Although studies on MOFs-based composite scaffolds in nerve tissue engineering are not as common as those in bone or skin tissue engineering, they have already shown exciting progress. A novel self-powered nerve conduit was fabricated by introducing UIO-6 and reducing graphene oxide (rGO) nanoparticles into PCL [199]. UIO-66 is a piezoelectric crystal material that can provide continuous endogenous electrical stimulation, and rGO has good electrical conductivity. Thus, the introduction of UIO-66 and rGO provides a bioadaptable neural interface to improve the oxidative metabolism and phenotypic switching of infiltrating macrophages in the damaged neural microenvironment, ultimately promoting nerve regeneration. As shown in Figure 5B, a ZIF-8 nanoparticle delivery system loaded with miR-29a (miR-29a@ZIF-8) was constructed to functionalize SF/gelatin-tylamine (SF/GT) composite hydrogel conduits [200]. The miR-29a@ZIF-8-loaded SF/GT hydrogel conduit significantly promoted the myelination of Schwann cells and the neuronal differentiation and axonal elongation of PC12 cells (Figure 5C). This study also showed that the p65 protein was transferred from the nucleus to the cytoplasm of macrophages after coculture with the miR-29a@ZIF-8 hydrogel. The expression level of inflammatory genes such as NOS_2_, TNF-α and IL-6 was significantly downregulated, and the expression level of the anti-inflammatory gene ARG-1 was significantly upregulated compared with that in the SF/GT hydrogel. This indicated that the addition of miR-29a@ZIF-8 could promote the polarization of macrophages from the M1 phenotype to the M2 phenotype and effectively regulate the immune microenvironment. Notably, the ZIF-8-incorporated hydrogel had the same gene expression changes as the miR-29a@ZIF-8-loaded hydrogel when compared to the gel group. Zn^2+^ may play a role in regulating the immune microenvironment. Therefore, this study not only demonstrated the potential application of ZIF-8 as a carrier to construct a miR-29a delivery system for peripheral nerve repair but also implied the potential of ZIF-8 nanoparticles themselves in peripheral nerve repair. In our recent work, we used an *in situ* loading approach to combine ZIF-8 nanoparticles with guided microfibers for sciatic nerve repair [201]. Histological evaluations confirmed that the introduction of ZIF-8 nanoparticles facilitated peripheral nerve regeneration. Recently, a self-powered enzyme-linked MN nerve conduit was fabricated [202]. Half of these MNs were loaded with ZIF-8 encapsulated with glucose oxidase, and half were loaded with ZIF-8 encapsulated with horseradish peroxidase. This nerve conduit generates microcurrents through an enzymatic cascade reaction that stimulates the regeneration of muscles, blood vessels and nerve fibers innervated by the sciatic nerve, ultimately accelerating the repair of sciatic nerve damage. It is clear that this work provides a novel approach to the treatment of sciatic nerve injury.

Zhai *et al*. [203] loaded a β-cyclodextrin metal-organic framework (CD-MOF) into a MN to deliver methylprednisolone sodium succinate (MPSS) to the SCI site in a controlled manner through the dura (Figure 5D–F). This work provides a new method for the drug treatment of spinal cord injury. Functional biomaterials based on MOFs have been less studied in nerve tissue engineering, especially *in vivo*; however, the excellent performance of functional biomaterials based on MOFs in bone and wound repair, as well as the great potential of MOFs nanoparticles in nerve repair, indicate that these materials have a great future in nerve tissue engineering.

### Cardiovascular

**Figure 6.**
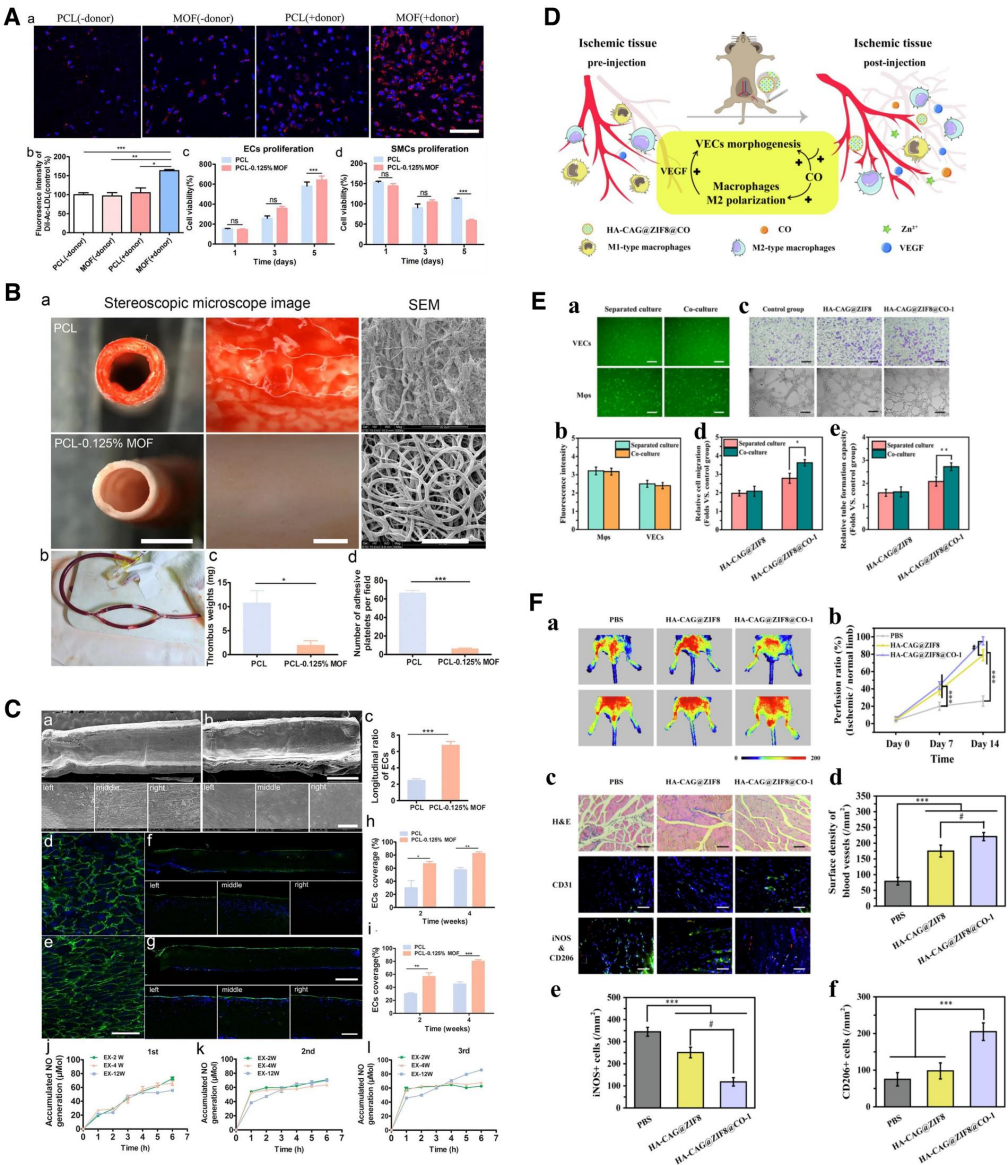
(**A**) The effect of PCL-MOFs scaffolds on HUVECs and SMCs function *in vitro*. (**B**) Evaluation of the hemocompatibility of vascular scaffolds by AV shunting in rats for 2 h. (**C**) *In situ* endothelialization on PCL-0.125% MOFs scaffolds. (**A–C**) Reproduced from Ref. [208] with permission of Elsevier, © 2021. (**D**) Schematic illustration of HA-CAG@ZIF8@CO regulating the dialog between vascular ECs and macrophages to promote vascular remodeling. (**E**) Dual cellular regulation of HA-CAG@ZIF8@CO under coculture conditions. (**F**) Blood perfusion and histopathology of ischemic hindlimbs. (**D–F**) Reproduced from Ref. [209] with permission of Elsevier, © 2023. （*p < 0.05, **p < 0.01, ***p < 0.001, #p< 0.05）

Cardiovascular disease is one of the most prominent refractory diseases worldwide. Its high morbidity, mortality and treatment cost impose a heavy burden on society and its patients [204, 205]. The application of MOFs in cardiovascular diseases can be divided into two aspects: drug-eluting stent coating and functional tissue engineering of blood vessels [206, 207]. Cu-based MOFs are most widely used in the cardiovascular field because Cu ions have excellent catalytic ability to generate nitric oxide (NO) from endogenous S-nitrosothiols (RSNOs) [207]. For instance, copper-based MOFs can be used as a component of an antithrombotic coating for cardiovascular implants. M199 loaded in PCL electrospun fibrous scaffolds can maintain long-term NO catalytic activity, significantly promote EC migration, increase the uptake of acetylated low-density lipoprotein (Ac-LDL) and inhibit platelet adhesion and activation (Figure 6A–C) [208]. In recent years, non-copper-based MOFs have been gradually applied in the cardiovascular field [209, 210]. The Fe-based MOF MIL-101(Fe) can be used as a mechanical enhancer, sustained-release drug carrier and MRI contrast agent to modify PCL-based vascular scaffolds [210]. MIL-101 (Fe)-modified artificial vascular patches could inhibit blood coagulation and promote vascular endothelialization [210]. ZIF-8 nanoparticles have been used to deliver carbon monoxide and coated with HA-CAG peptide to enhance its bioactivity [209] (Figure 6D). The nanoparticles can simultaneously target and regulate vascular ECs and macrophages and enhance the interaction between them, thereby promoting angiogenesis (Figure 6E and F). Currently, nanoparticles have only been used to treat critical limb ischemia, but this study introduces new horizons for angiogenesis therapy and has the potential to be applied to the construction of tissue-engineered blood vessels.

### Other tissues

Composite scaffolds based on MOFs have been used less frequently in other tissues. Since MOFs-based scaffolds have shown strong potential for application in the field of tissue engineering, researchers have made some attempts to use them in various tissues. For the first time, the potential of ZIF-8 to be incorporated into renal scaffolds while retaining its ability to remove uremic toxins has been demonstrated [211]. ZIF-8-reinforced bioinks can be used to print ear-shaped composite hydrogel constructs [138]. Glycidyl methacrylate-modified Uio-66-NH was used as an alternative coupling agent to improve the mechanical properties of dental fillers [212]. In addition, NO-loaded MOFs incorporated into PCL/gelatin-aligned coaxial scaffolds are used for tendon repair [131].

## Conclusion and future outlook

At present, the preparation of MOF-based functionalized biomaterials mainly involves solution blending and *in situ* growth methods. Increasing the variety of available preparation methods will facilitate expanding the application field of these materials. To date, the applications of MOF-based functional biomaterials have focused mainly on wound healing and bone repair because the antibacterial function of MOFs is the most prominent, and the probability of infection in these two types of injury is high. However, previous studies have suggested that MOFs also promote regeneration and angiogenesis and inhibit inflammation, among other functions, which suggests that MOFs have great research value in other areas of tissue engineering. Therefore, the application of MOFs-based functional materials in tissue engineering should be expanded. There are many types of MOFs with different functions, but only a few are used in tissue engineering, mainly ZIF-8, PCN and UiO-66. More types of MOFs should be investigated. In addition, to address the complexity of the regeneration of injured tissue, multifunctional scaffold materials will be needed. Hybridized or modified multifunctional MOFs are more promising for such applications. Furthermore, studies on the internal mechanisms of the important functions of MOFs in tissue engineering are rare. Therefore, future development directions may include the design of multifunctional MOFs, the application of MOFs-based composite scaffolds in other fields of tissue engineering, and the mechanistic study of the related functions. Although MOFs have been extensively studied for use in tissue engineering, much progress is still needed to enable their clinical translation. The safety evaluation of long-term implantation of MOFs-based scaffolds, comprehensive exploration of the mechanisms of action and establishment of relevant product standards are all urgently needed.
